# A pre-screening FISH-based method to detect CRISPR/Cas9 off-targets in mouse embryonic stem cells

**DOI:** 10.1038/srep12327

**Published:** 2015-07-24

**Authors:** Marianna Paulis, Alessandra Castelli, Michela Lizier, Lucia Susani, Franco Lucchini, Anna Villa, Paolo Vezzoni

**Affiliations:** 1 Milan Unit, Istituto di Ricerca Genetica e Biomedica, Consiglio Nazionale delle Ricerche, Milan, Italy; 2 Humanitas Clinical and Research Center, Rozzano, Milan, Italy; 3 Catholic University of the Sacred Heart, Cremona, Italy

## Abstract

The clustered regularly interspaced short palindromic repeat (CRISPR)/associated 9 (Cas9) technology has been recently added to the tools allowing efficient and easy DNA targeting, representing a very promising approach to gene engineering. Using the CRISPR/Cas9 system we have driven the integration of exogenous DNA sequences to the X-linked *Hprt* gene of mouse embryonic stem cells. We show here that a simple fluorescence *in situ* hybridization (FISH)-based strategy allows the detection and the frequency evaluation of non-specific integrations of a given plasmid. FISH analysis revealed that these integrations do not match the software predicted off-target loci. We conclude that the frequency of these CRISPR-mediated off-target DNA cuts is negligible, since, due to the occurrence of spontaneous double-strand breaks, we observed more aspecific plasmid integrations than those corresponding to predicted off-target sites.

CRISPR/Cas9 system is an innovative, specific and efficient approach to gene editing^1^,^2^,^3^. However, its application to the human situation needs a further investigation into possible additional modifications at other genomic loci (so called “off-target” events)^4^,^5^,^6^. Several methods have been used to minimize the degree of off-target events and to verify off-target non-specific cuts^7^,^8^,^9^,^10^,^11^,^12^. The current procedure to investigate on- and off-target events includes the analysis of the cell pool using nucleases able to recognize a mismatch in the double strand DNA (*in vitro* DNA cleavage assays), usually performed on a cancer cell line^13^,^14^. If the chosen single-guide RNA (sgRNA) performs well in this assay, the procedure is repeated on the desired cell line and targeted isogenic clones are identified by amplification and sequencing of the pertinent region. These steps allow the identification of the clones with the expected modification. However it is more difficult to exclude off-targets events: to this end, putative off-targets are analyzed by software scanning the entire genome for sequence similarities, and the putative loci are amplified and sequenced. Even in this case, off-targets not predicted by software tools cannot be identified, unless whole genome sequencing (WGS) of both the parental and the modified clone is performed. Two recent papers reported the results obtained performing WGS in targeted pluripotent stem cell clones demonstrating a low frequency of off-target events^15^,^16^. However, this approach is expensive and time consuming. Therefore, a procedure allowing quick, although less precise, identification of the relative frequency of both on- and off-targets could be helpful in the initial phase of the analysis, where one among several possible sgRNA is designed, allowing the elimination of candidates with lower ratio between on- and off-target events.

Here we demonstrate that fluorescence *in situ* hybridization (FISH), a cytogenetic technique widely used for diagnostic applications but also for cytogenetic and genome research^17^,^18^,^19^, is a good tool to check the result of gene editing using the CRISPR/Cas9 approach. This assay can be applied in the initial step of analysis and can be directly performed on the cell line that has to be targeted (Fig. 1).

## Results and Discussion

### Functional testing

**Figure 2:**
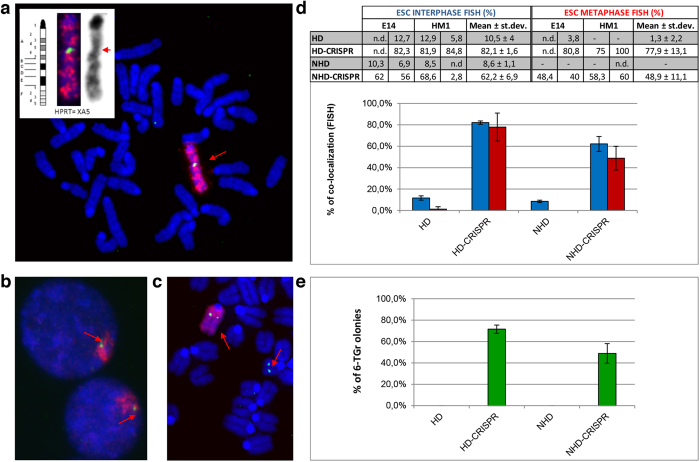
Analysis of ESC lines transfected with CRISPR vector targeting the *Hprt* locus. Representative FISH images performed with whole mouse X chromosome painting (red) and NHD DNA (green) probes, showing: (**a**) co-localization in a metaphase spread (insert shows the X chromosome ideogram, the enlarged section of the metaphase with the hybridized and the inverted-DAPI X chromosome respectively; *Hprt* locus on the XA5 band is indicate with the red arrow); (**b**) co-localization in interphase nuclei, and (**c**) an extra-integration in addition to the correct one in the *Hprt* locus in a metaphase spread. All chromosomes and nuclei were counterstained with DAPI (blue); red arrows indicate the localization of the NHD DNA probe. (**d**) Interphase FISH and metaphase FISH analysis. The table and the histogram show the percentage of whole mouse X chromosome painting and NHD DNA probe co-localization in interphase (blue) and metaphase FISH (red) performed on the neomycin resistant pools. (**e**) Survival rate of neomycin resistant colonies after 6-TG treatment (6-TG^r^) (green, n = 3). HD: Cells transfected with the homologous donor (HD) plasmid; HD-CRISPR: Cells transfected with both CRISPR and HD plasmids; NHD: Cells transfected with the non-homologous donor (NHD) plasmid; NHD-CRISPR: Cells transfected with both CRISPR and NHD plasmids.

To evaluate the applicability of FISH as an appropriate approach to test the functionality of the chosen sgRNA, we performed targeted experiments in two murine embryonic stem cell (ESC) lines, E14 and HM1, both with a normal karyotype (40, XY). The former is one of the first ESC lines established; the latter is a *hypoxanthine-guanine phosphoribosyltransferase* (*Hprt)* deficient derivative of E14^20^. The *Hprt* locus is localized on the XA5 band of the mouse X chromosome (Fig. 2a, ideogram) and its inactivation confers resistance to 6-thioguanine (6-TG).

Using the Optimized CRISPR Design (OCD) tool (http://crispr.mit.edu/), we designed two sgRNAs (denominated 2m and 3m respectively) targeting the *Hprt* locus in a region common to both E14 and HM1. We used two CRISPR constructs that cut the *Hprt* gene at two different sites since a different locus could not be verified by such a simple and fast assay as 6-TG selection.

The two sgRNAs were cloned into the Cas9/sgRNA pX330 vector (hereafter the CRISPR2m or CRISPR3m vector respectively)^2^,^13^,^21^,^22^,^23^ and verified by sequencing. The CRISPR2m vector was transfected in the ESC lines together with a DNA fragment carrying a neomycin resistant gene either flanked or not, on both sides by a region of homology to the targeted *Hprt* region, hereafter denominated “homologous donor” (HD) or “non-homologous donor” (NHD) plasmids, respectively. In addition, the same DNA plasmids were transfected without the CRISPR vector. HM1 and E14 cells were selected with neomycin. Since the *Hprt* locus in HM1 is already inactivated, 6-TG selection cannot be used to identify targeted clones in this ESC line, while inactivated clones from E14 can be isolated by this strategy^24^.

After seven days, FISH experiments using the mouse X chromosome painting and the NHD DNA probes were performed on the pooled cells. Interphase FISH analysis of the E14 and HM1 pools showed co-localization of the two probes (Fig. 2b). As expected, the presence of the CRISPR vector greatly increased the targeting of the HD plasmid at the *Hprt* locus from about 10.5% to 82.1% (Fig. 2d, blue bars). The NHD plasmid integrated specifically at similar, albeit lower, levels (62.2%), indicating that the availability of a DNA break is the most important factor in the integration process of exogenous sequences. FISH metaphases were also examined showing the correct localization of the NHD probe to the *Hprt* gene and confirming the data obtained in the interphase nuclei (Fig. 2d, red bars). Similar results were obtained when CRISPR3m vector was used (co-localization with HD-CRISPR3m: 72.2%; with NHD-CRISPR3m: 50.8%).

### 6-TG selection

FISH results were also supported by 6-TG selection data. In three parallel experiments, neomycin resistant E14 pooled cells were selected in 6-TG medium; concomitant resistance to 6-TG treatment indicates that the *Hprt* locus of E14 neomycin resistant clones has indeed been inactivated. We observed that, upon HD-CRISPR treatment, about 70% of neomycin-resistant E14 colonies survived 6-TG selection (Fig. 2e, green bars). Since the selection in 6-TG is indicative of functional inactivation of the *Hprt* gene by precise targeting, either HD or NHD, the results obtained show that most insertions detected on the X chromosome represent true targeting events, giving an easy quantification of the efficiency of the correct targeting. This suggests that detection of a donor plasmid at endogenous targeted loci by FISH could give an estimate of the frequency of targeted events. Moreover, this approach is more immediate and simple with respect to other molecular approaches such as Southern blot performed on the cell pool which would only detect recurrent integrations at the same chromosomal locations. Hence, in this specific case, although the numerous integrations at the on-target site would be detected, all off-target integrations occurring rarely in several different regions would be below the sensitivity of the Southern. Similar results were obtained when the selection was performed on CRISPR3m-transfected neomycin resistant pools (HD-CRISPR3m: 65%; with NHD-CRISPR3m: 52.6%). Therefore this simple assay might give a rough idea of the efficiency of the chosen sgRNA.

### Analysis of integrations at non-targeted loci

**Figure 3:**
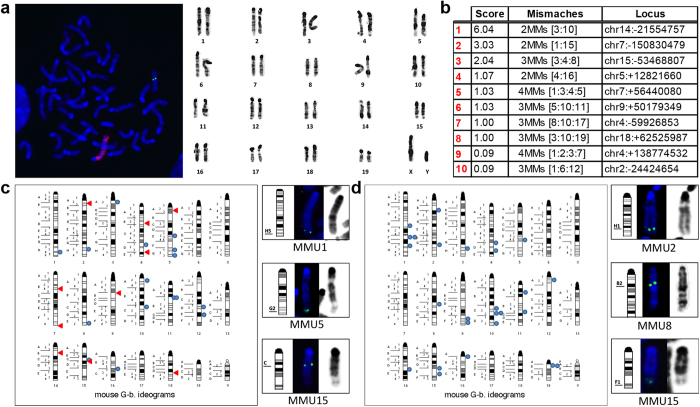
Analysis of off-target events. (**a**) Representative metaphase FISH and the corresponding inverted-DAPI banding karyotype. (**b**) List of the first 10 off-target sequences predicted by Optimized CRISPR Design tool relative to the chosen sgRNA; their mismatched bases (number and position) and chromosome localization are indicated. (**c**) Mouse chromosomal ideogram showing the 16 cases where the NHD DNA probe was mapped on chromosomes other than the X chromosome in the HD-CRISPR pool (Total metaphases scored n = 100). Red arrows indicate the localization of the predicted putative off-target sites; blue circles indicate the real localization of the NHD plasmid. Three examples of chromosome mapping other than X chromosome are shown. (**d**) Mouse chromosomal ideogram showing the 26 cases where the NHD DNA probe was mapped on chromosomes other than the X chromosome in the HD pool (Total metaphases scored n = 26). Blue circles indicate the localization of the NHD plasmid. Three examples of chromosome mapping are shown.

FISH approach could directly be performed on metaphase spreads of the cell line of interest. The analysis of the DAPI banded metaphases allows the identification of possible gross chromosome abnormalities occurring during the procedure as well as the chromosome localization of the vector integration. In this way, the frequency of predicted target events, with respect to off-target events can be evaluated, before proceeding to further investigations (Fig. 3a).

Our assay also shows cells bearing the donor plasmid integrated on other loci in addition to the specific target site (Figs 2c and 3a). These dual (or possibly extra) events would not have been identified with sequencing of predicted off-target sites^14^. In addition, a rough evaluation of the occurrence of predicted off-target events could be obtained if the FISH signal localized to a specific chromosomal band with an elevated frequency. Interestingly, when the FISH signals in the 16 of 100 analyzed metaphases (16%) in which the integration occurred outside of the *Hprt* chromosomal band were mapped, only one possible co-localization with the top ten off-target sites predicted by the OCD software was detected (with a predicted sequence containing 3 mismatches; see Fig. 3b,c). The localization of the FISH signals was performed only in the metaphases with satisfactory DAPI-G like banding relative to chromosome bands. We also analyzed the chromosomal localizations of the NHD probe in transfection without the CRISPR vector (Fig. 3d) and we did not observe significant overlapping localization sites, as expected by random integrations.

It is generally accepted that a non-viral NHD plasmid inserts randomly into the genome in locations where a double strand break (DSB) has occurred by chance^25^. The insertion events identifiable by FISH are clearly only a fraction of the occurring DSBs, therefore only a subset of spontaneous or nuclease induced DNA cuts can be detected by our assay. However, we argue that if CRISPR-related off-target DNA cuts occurred at a relevant frequency, a bias should be detected by our assay. We also isolated 10 clones from one experiment and amplified and sequenced the three top predicted off target sites, confirming that no indel was present. At the same time, our data suggest that off-target cuts due to CRISPR could be negligible when the total number of spontaneously occurring, probably unavoidable DSBs, are taken into account. These events will go undetected unless whole genome sequencing is performed. Altogether, our results show that FISH analysis could give a general and simple appraisal of the efficacy and specificity which could usefully complement classical molecular analysis of CRISPR targeting.

In conclusion, this assay represents a promising approach to obtain a quick evaluation of the behavior of the candidate RNA guides, to evaluate off-target events and to identify clones bearing additional insertions outside the chosen locus, including those not predicted by sequence similarities. We propose to use this cytogenetic approach in the preliminary evaluation of the candidate sgRNA guides, thus reducing the time and effort linked to a late elimination of clones with unacceptable modifications.

## Methods

### CRISPR construct

Two sgRNA oligo sequences: 2m-CRISPR-HPRT-EX3 (F: 5′-CACCGTGGCCCTCTGTGTGCTCAAG-3′; R: 5′-AAACCTTGAGCACACAGAGGGCCAC-3′) and 3m-CRISPR-HPRT-EX3 (F: 5-CACCGAGCCCCCCTTGAGCACACAG-3; R: 5-AAACCTGTGTGCTCAAGGGGGGCTC-3) were designed using the Optimized CRISPR Design (OCD) tool (http://crispr.mit.edu/). The oligos were annealed and ligated to the pX330 vector (Addgene) previously digested with *Bbs*I (New England Biolabs).

### Analysis of Off-Target Sites by PCR and sequencing

The off-target analysis was performed by PCR and sequencing of the amplicons surrounding the predicted off-target sites with the higher score. The sequences were amplified from DNA extracted from individual CRISPRm2-transfected neomycin resistant HM1 ESC clones. For each clone, the top three potential off-target sites were analyzed. PCRs were performed using specific primer pairs (see Table 1). PCR reactions were performed under the following conditions: initial denaturing for 5 min at 94 °C; denaturing for 30 sec at 94 °C, annealing for 30 sec at 57 °C, extension for 30 sec at 72°C, repeated 30 times; final extension for 5 min at 72°C. PCR products were purified, quantified and sequenced. Multiple alignment of the DNA sequences, were performed using the ClustalW2 algorithm.

### pPNT-HPRT construct

The 5′ and 3′ genomic regions of HPRT gene surrounding the sgRNA target were amplified and cloned into pPNT vector (NHD). The pPNT-HPRT construct (HD) contains the TK (thymidine kinase) gene from herpes simplex virus and the neomycin selectable marker, flanked by the 5′ and 3′ genomic regions of about 800 bp homologous HPRT arms.

### Cell cultures, transfection and selection

E14 and HM1 ES cells were cultured on gelatin-coated plates with standard ES cell culture conditions.

Before transfection the cells were seeded into 6 well plates (approximately 5 × 10^5^ cells per well). The transfections were performed using Lipofectamine 2000 (Life Technologies) according to the manufacturer’s protocol with CRISPR (2 μg); pPNT-HPRT (2 μg), CRISPR and pPNT or pPNT-HPRT constructs (2 μg + 2 μg). The medium was changed after 6 h.

The neomycin selection (350 μg/ml) (Sigma) was added 24/48 h after transfection and the resistant cells were pooled and analyzed by FISH.

The E14 neomycin resistant cells were seeded at a low density (1 × 10^4^ cells/well) and subjected to neomycin or neomycin and 6-TG media (30 μM) (Sigma Aldrich). After 3–4 days the resistant colonies were counted.

### FISH

FISH experiments were carried out on interphase and metaphase cells from transfected ES cells as previously described^26^. Briefly, cell cultures were treated with KaryoMAX colcemid (Life Technologies) at a final concentration of 0.1 μg/ml for 2 h at 37 °C and cells were then detached by treatment with 0.25% trypsin/ EDTA (Lonza). After hypotonic treatment with 0.075 M KCl and fixation in methanol:acetic acid (3:1 v/v), the cell suspension was dropped onto a slide and air dried. Slides were treated with 0.004% Pepsin (Sigma) at 37 °C for 30 sec and dehydrated through the ethanol series before denaturation in 70% formamide/2xSSC.

The NHD DNA vector (pPNT) and the red labeled whole chromosome painting probe (WCP), specific for the mouse X chromosome (Applied Spectral Imaging) were used as DNA probes. The pPNT probe was labeled via nick translation (Life Technologies), using Bio-11-dUTP (Roche) and resuspended in hybridization buffer (50% formamide, 10% dextran sulphate, 1x Denhart’s solution, 0.1% SDS, 40 mM Na2HPO4 pH 6.8, 2xSSC) containing 10x mouse Cot1 DNA (Life Technologies).

Before hybridization the probes were denatured at 80 °C for 10 min and pre-annealed at 37 °C for 20 min. Hybridization was carried out overnight at 37 °C. Stringent washings were performed in 50% formamide/2xSSC at 42 °C. For biotin detection the slides were incubated with FITC-conjugated avidin DCS (Vector Laboratories), then with biotin-conjugated anti avidin D antibody (Vector Laboratories) and finally with FITC-conjugated avidin DCS. Avidin and all the antibodies were used at a final concentration of 5 μg/ml.

Slides were mounted in Vectashield mounting medium with DAPI (Vector Laboratories), and then were scored under an Olympus BX61 Research Microscope equipped with a cooled CCD camera. Images were captured and analyzed with Applied Imaging Software CytoVision (CytoVision Master System with Karyotyping & FISH). To identify individual chromosomes and to assign the location of signals to specific chromosome regions, inverted digital images of DAPI banded chromosomes were used. For each neomycin resistant pool at least 20 metaphases and 200 interphase nuclei were analyzed.

## Additional Information

**How to cite this article**: Paulis, M. *et al.* A pre-screening FISH-based method to detect CRISPR/Cas9 off-targets in mouse embryonic stem cells. *Sci. Rep.* **5**, 12327; doi: 10.1038/srep12327 (2015).

## Figures and Tables

**Figure 1:**
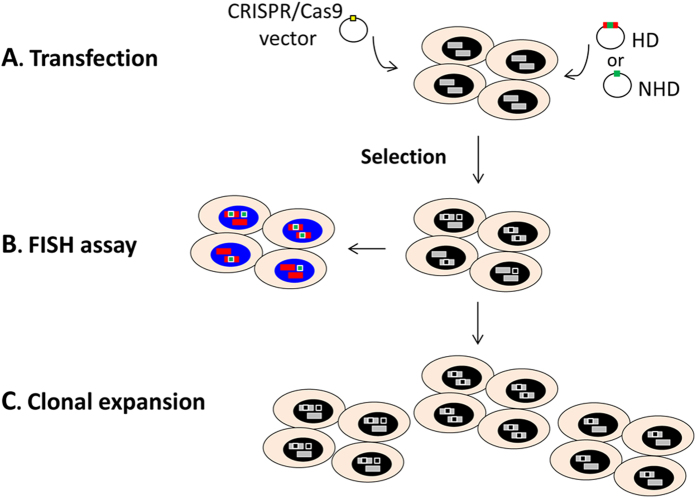
Schematic outline of the FISH approach to the CRISPR procedure evaluation. In the first step, the cell line is transfected with the CRISPR/Cas9 (carrying *S*.*pyogenes* Cas9 and its sgRNA) and the donor vectors carrying a selectable marker (either HD: Homologous Donor; or NHD: Non Homologous Donor); subsequently a selection is applied. In the second step FISH is performed on antibiotic-resistant cell pool to check the genome localization of the donor vector; the co-localization of two probes (targeted region in red and the exogenous sequence in green) indicates the correct targeting. Lastly, on the basis of the FISH result, the pool is expanded and clones of interest are isolated.

**Table 1:**
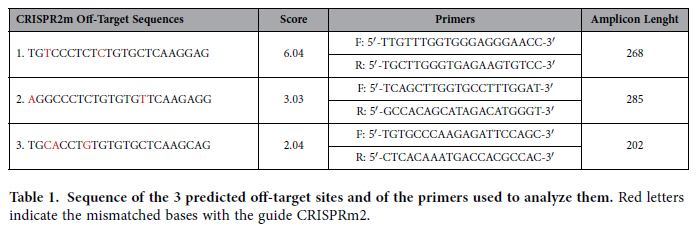
Sequence of the 3 predicted off-target sites and of the primers used to analyze them.
